# Barriers to venipuncture-induced pain prevention in cancer patients: a qualitative study

**DOI:** 10.1186/s12904-016-0180-x

**Published:** 2017-01-17

**Authors:** Marilène Filbet, Philip Larkin, Claire Chabloz, Anne Chirac, Léa Monsarrat, Murielle Ruer, Wadih Rhondali, Cyrille Collin

**Affiliations:** 1 Department of Palliative Care, Centre Hospitalier de Lyon-Sud, Hospices Civils de Lyon, 165 Chemin du Grand Revoyet, 69310 Pierre-Bénite, France; 2 UCD School of Nursing, Midwifery and Health Systems & Our Lady’s Hospice and Care Services University College Dublin, Stillorgan Rd, Belfield, Co., Dublin, Ireland; 3 Coordination for the Evaluation of Professional Practices in Healthcare in the Rhône-Alpes Region, 162 Avenue Lacassagne Bâtiment A - 7ème étage, 69424 Lyon Cedex 03, France; 4 Lyon 2 University, Psychology Institute, 5 Avenue Pierre Mendès France, 69500 Bron, France

**Keywords:** Procedural pain, Cancer, Prophylaxis, Nursing, Incidental pain

## Abstract

**Background:**

Procedural pain reduces the quality of life of cancer patients. Although there are recommendations for its prevention, there are some obstacles for its management. The purpose of this study was to analyze the barriers to procedural pain prophylaxis in cancer patients reflecting the views of the nurses.

**Methods:**

We used qualitative methodology based on semi-structured interviews conducted with nurses, focusing on practices of venipuncture-induced and needle change for implantable central venous access port (ICVAP) pain management in cancer patients. A thematic analysis approach informed the data analysis.

**Results:**

Interviews were conducted with 17 nurses. The study highlighted 4 main themes; technical and relational obstacles, nurses’ professional recognition, the role of the team, and organizational issues. Participants understood the painful nature of venipuncture. Despite being aware of the benefits of the anesthetic patch, they did not utilize it in a systematic way. We identified several barriers at different levels: technical, relational and previous experience of incident pain. Several organizational issues were also highlighted (e.g. lack of protocol, lack of time).

**Conclusions:**

The prevention of venipuncture-induced cancer pain requires a structured training program, which should reflect the views of nurses in clinical practice.

## Background

Managing cancer pain is a public health issue in France [1] but awareness of the frequency and impact of incident pain, as well as the necessity for prevention is more recent [2, 3]. Of note, specific recommendations to address this have been proposed [2, 3]. Incident pain is defined as an acute pain, caused by a therapeutic intervention or planned care action, which can be prevented through adapted measures [2, 3]. Incident pain, even of moderate intensity, is nonetheless challenging as it is often associated with an activity, such as movement and is therefore repeated and consistent. The repetition of the procedure favors a secondary hyperalgesia related to sensitization of the central nervous system [3–7]. Pain results from an increase in hyper excitability with a response to nociceptive stimulus of a greater intensity and duration, while reducing the response threshold to a nociceptive stimulus. No nociceptive stimulus should thus be considered as benign as all stimuli will, in the long run, affect the central nervous system. This sensitization can be prevented through analgesic prophylaxis [3]. Care-induced (procedural) pain is very common, noted in up to 60% of cases, depending on the procedures. Couteaux studied the prevalence and intensity of care-related pain in adult patients hospitalized in various departments and showed that the most frequent painful procedures were venipuncture and mobilization [8]. Regardless of the population (adults, children), care-induced pain is under evaluated and under treated [9]. The consequences of unaddressed incident pain can shift from stressful recall to deterioration in the patient's quality of life, leading to greater overall suffering [3]. Although there are several papers exploring the epidemiology of procedural pain and its prevention in cancer patients, as analgesic, sedative, topical local anesthetic agent and non-pharmacological methods, there is limited information regarding the obstacles for its management.

This study aimed to explore and describe the barriers to the appropriate and systematic use of analgesia to relieve incident pain in 2 types of procedures (venipuncture and needle change for ICVAP) from the viewpoint of nurses in clinical practice.

## Methods

This is a qualitative exploratory study. We chose to use a qualitative design, as we wanted to address nurses’ perceptions about a complex area that is not easily quantifiable. The study was approved by the local ethics committee and the Institutional Review Board at the Hospices Civils de Lyon.

### Context

The study was conducted in one Academic Medical Center. In our institution, recommended prophylaxis for this type of care is the use of a lidocaine-prilocaine patch, but there is no established protocol.

#### Participants

A purposive sample of nurses was recruited from 18 participating departments. These 18 units were medical or surgical departments managing adult cancer patients. Inclusion criteria were that participants should be a nurse in current clinical practice and have responsibility for the care and management of cancer patients. Written information about the study and its purpose was given to all participants. Written informed consent was signed before enrollment in the study.

#### Data collection

##### Nurses and practice characteristics

Biographic and demographic data were collected during the interviews age, gender, clinical setting, number of years in practice and number of years in this department, and if they were the pain resource nurse of their unit. The pain resource nurse is in charge of applying institutional protocols regarding pain issues, organizing training sessions and all quality improvement initiatives. We also asked them to provide the number of venipuncture and needle change for ICVAP per week in their department, and how many were performed with a prophylactic approach to pain management (use of a lidocaine-prilocaine patch or equivalent).

#### Analysis

Following an introductory meeting, an individual face-to-face semi-structured interview was conducted with each nurse by a researcher skilled in qualitative research methods (WR), in a quiet room. Interview questions were geared towards eliciting open-ended responses to acquire specific information about the nurses’ thoughts associated with procedural pain, especially regarding venipuncture and needle change for ICVAP. In our study, we conducted two preliminary interviews to test the quality of the questions (ie understanding and accuracy) we had planned to use (Table 1). All interviews were digitally audio-recorded and then fully transcribed. The name and personal information of participants were removed from transcripts, and code numbers were assigned. These interviews were subjected to a thematic analysis informed by Grounded Theory applied to the data to extrapolate results. Grounded theory enables theory to emerge inductively from data [10]. The first step is open coding, which consists of multiple reviews of the transcripts to identify and categorize data [11]. Five authors (WR, CC, LM, MR, and MF) performed this first step independently. The second step divides the interview into "units of meaning" to highlight, in a third step, the underlying meaning of what the participants wanted to express. We then completed this open coding (i.e. analysis of each interview independently) using axial coding to connect interviews. Constant comparison of the interviews identified common elements and differences among the participants’ responses. During this last step, all the elements were categorized into major themes. The discrepancies in analysis were discussed during a meeting with the scientific committee. The first author (MF) performed the translations of the reported quotations.

**Table 1:** List of questions used during the semi directive interview

• What is your experience of venipuncture or needle change on central catheter?
• What did you feel when you make a venipuncture or a needle change on central catheter?
• How did you feel when you encountered difficulties in a venipuncture or a needle change on central catheter?
• How do you feel when the patient expresses pain in a venipuncture or a needle change on central catheter?
• How do you perceive your role as a nurse for the prevention of induced pain during venipuncture or needle change on central catheter?
• Could you specify the nursing actions (venipuncture or needle change on central catheter) in which it is necessary to conduct pain prophylaxis?
• Are there any situations where pain prophylaxis is complicated (feasibility, time, technology)?
• Are there situations where the patient refuses pain prophylaxis?
• How many venipunctures do you do during one week? How often do you perform pain prophylaxis?
• How many needle changes on central catheter do you make during one week? How often do you perform pain prophylaxis?
• Is there something you would like to add?

In qualitative research, data collection ends when none of the analysts recognize new or unique themes. This approach is known as data saturation, particularly reflective of Grounded Theory. In our study, data saturation was reached when transcripts from 17 semi-qualitative interviews had been coded. It took 4 months to recruit the 17 consecutive study participants.

This study follows the COREQ guidelines.

## Results

### Nurses’ characteristics (Table 2)

**Table 2:** Characteristics of the nurses and their practice (N = 17)

Characteristics	*N* (%)
Female, n (%)	16 (94)
Age, mean (SD)	34 (9)
Years of experience, mean (SD)	4 (3)
Pain resource nurse (%)	7 (41)
Number of venipunctures per week; mean (SD)	18 (10)
Number of pain prophylaxis for venipuncture per week; mean (SD)	2 (3)
Number of needle changes per week; mean (SD)	7 (5)
Number of pain prophylaxis for needle change per week; mean (SD)	5 (5)

SD; standard deviation

The population consisted of 17 nurses, with a mean age of 34 years (SD = 3), 16 of whom were women (94%), and 7 (41%) were the pain resource nurse of their department Venipuncture was a very common procedure (18 per week on average), more frequent than needle changes (twice a week in average). Pain prevention rates also differed: 11% for venipuncture and 71% for needle changes (ICVAP).

### Findings (Table 3)

**Table 3:** Findings

Theme	Positive	Barriers
Technical aspects	Good knowledge of procedural pain Skill Unlimited access to the patch Good knowledge of behavioural techniques Pain recognition	Delayed efficacity Multiple injection sites Vasoconstriction Emergency Denial or neglect of the pain
Professional recognition	Technical dexterity Professional identity Good relationship with the patient	Failure means nurse has failed Focus on the technical task Poor relationship with the patient
Team work	Help and support	Rivalry
Organizational aspects	Team culture/commitment Clear protocol established	No protocol established Workload

The interviews’ analysis highlighted 4 main themes: technical and relational aspects, nurse’s professional recognition, teamwork, and organization. Within these 4 themes, positive and negative/barriers were identified and will be described.

#### Technical and relational aspects

First, we explored the perception nurses had of pain induced by peripheral venipuncture and changing needle for ICVAP in adult cancer patients, from a technical point of view.

Most nurses said they found satisfaction in their work and had good experience of venipuncture and ICVAP needle changes. They reported having received good-quality technical training initially and seemed capable of adapting theoretical skills to the practical application of finding and accessing patient's veins. Further, the painful aspect of the repetitive procedure was clearly identified:

*"But they are given so many punctures. After a while, (…) it can only be painful"(N13)*

All nurses had a good clinical understanding of the analgesic patch, and of how to use it, agreeing that they had an unlimited access to lidocaine-prilocaine patches in their respective units.

One of the first obstacles to preventing pain with a lidocaine-prilocaine patch was the question of the area where it should be applied. Nurses separated the 2 procedures (i.e. venipuncture and needle change for ICVAP). For the needle change, the site is fixed, and the patch easy to apply, but for venipuncture the site varied depending on the quality of patient's veins, and it is sometime necessary to apply two or more patch in various places. Another technical limitation identified was related to time taken in procedure and the expectation of their organization. The action time of a patch (i.e. one hour from application to insertion of needle) was seen as too long, as it caused excess delay. Thus it was not always adapted to the practice of the department or in emergency situations. This time issue can be related to the need for an urgent intervention or, when it is necessary to quickly treat the patient:

*"When it must be done in emergency (…) a patient whose status is very bad, we have to set up a catheter, and then there is no time to wait for anesthesia."(N7)*

A few nurses reported a vasoconstrictor effect, limiting the visibility and access to the veins: *"For venipuncture and peripheral catheters, I don't use any patch ('…) I think it really fades away the veins" (N11)*

Moreover, nurses reported other physical or emotional conditions which might complicate the procedure: an anxious febrile patient, or someone in septic shock. Some nurses identified a certain level of neglect in preventing incident pain, or even a denial of its impact and consequences:

*"I don't propose patches to patients, that's true, because I don't think about it";* (N6)*"except for patients who are regularly given an injection, we know they don't hurt."(N7)*

The nurses decided *a priori* whether or not to apply the patch, depending on their assessment and did not propose it to every patient:

*“We know if they need a patch or not.” (N6)"The patient has to insist strongly with me to have a patch."(N8)*

Few nurses were clearly prejudiced against the patch:

*"We are afraid there might be a penetration. That's why we generally don't put patches during needle change."N6; «It must not be overused (…) it's not good to use it on the long run"(N7)*

Nurses were able to adapt their technique to the patient's status. They understood the importance of listening, of the patient's involvement, and of the different relaxation or behavioral techniques that could be applied.

It appeared that in some cases, nurses minimized the pain related to the procedure:

*“I blame myself a little less because it's a very temporary pain* “. (N6)

It would appear that the subjective nature of the patient's experience was used to deny the existence of incident pain. For example, in the medical oncology ward, the balancing of all the painful procedures compared to the supposed harmlessness of a venipuncture was noted:

*“They live so many things every day (…) all the invasive procedures (…) which hurt so much (…) that a catheter, it's… almost nothing. "(N6)*

Another point reported by nurses was that some patients were more sensitive than others:

*“I think it's related to the patient"; (N16)*

“This concept of "bad patient" also corresponded to a certain form of denial; the person responsible for the pain was no longer the one who gave the injection, but the one who received it, and who would respond badly to the nursing care:

*"They will react so that I feel uncomfortable”.( N4)*

#### Nurse professional recognition

There was also evidence that technical dexterity influenced relational aspects: a well-performed puncture improved the status of the nurse and brought unanimous professional recognition from colleagues.

*"If we clearly see it's going to be difficult (…) to give an injection to the patient, we are like "”ah a little technical difficulty", a little challenge (…) If we succeed, we are very proud"(N16)*

Technical success was described in terms of professional gratification, and the nurses identified themselves in this procedure:

*“We feel good about ourselves, well (…) you gave an injection once to the patient and you succeeded (…) well I am pleased with myself”.(N4)*

Further, it was proposed that the venipuncture procedure was seen as a definition of professional nursing identity:

*“It has to do with the efficacy of our profession"(N8)*

The identification of nurses with this technical procedure (venipuncture) made them raise doubt about their capabilities and professionalism when they experienced failure:

*“It kind of hurts when you think about it… you suck at your job, you cannot give an injection”*. (N4)

Dexterity was perceived as reassuring for patients and the absence of care-induced pain created a better relationship between nurse and patient. Failure to perform a venipuncture meant perceived failure as a professional nurse. Where failure occurred during first contact with a patient it was perceived to influence the on-going and future relationship with the patient:

*“Cannot manage to give an injection (…) it means we're not a good nurse. I think (…) it influences our relationship with the patient.* “(N16)

Seeking success at all costs revealed a tension between the discourse regarding a professional holistic nursing response to patient needs and the practical exercise of venipuncture. Patients were presented as diminished, reduced to their veins*:*

*“We’re going to put a needle, a tube into a vein.* “(N8)

To be able to focus on the technical act and perform it successfully, nurses explained the need to sometimes focus more on the task than the patient*.*

*”Sometimes we have to set the patient aside (…) so we have to ignore the pain to be able to continue the nursing care. »(N6)*

#### Teamwork

Nurses relied on and felt supported by teamwork. When they failed in the procedure, they could ask a colleague to take over, although the number of attempts made before deferring to a colleague was variable (between 1 and 3 attempts). Moreover, the presence of a team leader as well as a protocol to which they could refer was a reassuring element as it provides a support if needed. The team culture was also evoked as it enabled beginners to be supported in their practice by the most experienced professionals

*"It must be a team commitment" ;( N8)*

*"I am happy to supervise young nurses (…) who have just finished school and to show them how we do it" (N 15)*

Despite the positive aspects noted, the heterogeneity in nursing practice made delegation complex:

“*She put the patch here, so I don't see where to insert the needle, but I would do it on this one where there is no patch”.* (N8)

Some participants reported the existence of a rivalry between the nurses, regarding the skill (those who did it better that day) and regarding the patients (this patient prefers this nurse). *"She told me* [ the patient ] *could you ask your colleague who took my blood this morning, she's very talented"(N4)*

#### Organization

It was perceived that the existence of a protocol for everybody to follow could avoid the separation between nurses performing prevention with the patch and the others:

*"Doing a protocol to prevent pain in new patients, when we set up a catheter (…) if we do it, we won't be poorly looked upon by our colleagues saying "she's considered as the nice nurse, and I am the bad one" (N6)*

If a protocol was established, using a lidocaine-prilocaine patch was considered easier. Nurses were aware of the importance of anticipatory organization:

*"It's really too bad, because it's true we could anticipate a lot more concerning this care"; "the patients leave with a patch they have to put at home before arriving at the hospital, for instance for chemotherapies"*.(N5)

Although the delayed action time of the lidocaine-prilocaine patch was presented as a barrier, in some units, the nursing organization in relation to this was reviewed:

*"It's a false problem. (…) When we know there are… in the morning (…) needles or catheters to change, we have to organize ourselves (…) to put the patch and let another colleague in the afternoon insert the needle"*.(N17)

The presence of a protocol was reported as useful only if it belonged to the department (i.e. if it has been developed with the nursing staff of the department and was adapted to the department's logistical constraints).

Further, when nurses could use a patch without medical prescription, they proposed the patch systematically if it was indicated on a prescription and more randomly if there was no prescription.

The role of the physicians in prescribing and repeating painful procedures was sometimes noted:

*“The physicians, with them, it's every day, (…) they don't check who we gave an injection to the day before, they prescribe again and that's it".(N16)*

Lack of time and workload was reported as a limit to preventing induced pain:

*“Well, in the morning, when we have to give an injection to 8 patients, find the vein, put the patch to everyone, come back later, it's impossible! “.(N10)*

## Discussion

This qualitative study highlights the gap between the knowledge about procedural pain and an under use in clinical practice. Four points need to be discussed, training and experience, the perceived professional role of nurse, organizational aspects and patient information.

In our study, the population consisted of nurses, with an average age of 34 years, with a moderate 4 years post-qualification experience. The content of nurse training has changed over the last 15 years, emphasizing the importance of pain and its management [12]. This population was thus representative of the current training provided.

Although the nurses reported a robust professional nurse-training program, it seems that this was insufficient to sensitize nursing staff to pain management. Our results are consistent with the literature [13]. Our results also suggest that pain prophylaxis was influenced by professional experience such as practice in an oncology ward [14]. This study supports previous studies noting gaps regarding the knowledge of analgesics [15–18]. This last point can be explained by the discrepancies of the nurses' seniority. We also noted a perceived trivialization of pain for some procedures. The reasons proposed is that the perception of patients' pain by nurses is different than the patient's own regarding their own pain, especially for venipuncture [19].

In our results, the multiplicity of the puncture sites is reported by the nurses as a real challenge to preventing pain during venipuncture compared to needle changes. This limit was not found in the literature and thus it seems very important to include this kind of limitation into the development of future protocols.

The qualitative analysis also highlighted concerns related to the nurses’ professional practice.

First, nurses consider themselves as technicians [20] and identified themselves with the skill of the technical procedure. A lack of dexterity led to pain [2, 21], which would at the same time call into question the relational link to best nursing practice [22]. In fact, if this procedure failed or was painful, the patient could doubt the nurse's professional skills or competency, or even be hostile towards the nurse [2]. Regarding the reported difference about pain sensitivity between patients, this may be interpreted as a defensive mechanism (i.e. incident pain does not come from a lack of skills or dexterity, but rather from an external reason) [22]. Similar results were found in a study based on interviews undertaken with health care assistants and nurses about pain induced by terminal care [23].

Nurses reported difficulties related to the organization. A re-focus of all the nursing staff and physicians practice and procedure would be necessary to organize nursing care in order to enable more systematic prevention [6, 12, 20]. This should be done through the nurses' training and participation in writing protocols [20]. While preventing painful procedures is a major part of the nurses' role, prevention in practice would appear to be more frequent when there is a protocol, i.e. referring to their prescribed role.

The barriers identified in this study will enable us to adapt the content of training sessions for the nurse about the pain management induced by venipuncture and ICVAP needle changes. The program should consist of cognitive and organizational elements, along with a reflective process on the nurses' expectations and perspectives.

Finally, it could also be important to inform patients about care-induced pain prevention. Some studies reported a resignation from the patients facing pain in a hospital. It is seen as part of their affliction and as a compulsory transition towards recovery [2, 24]. We propose that patients should receive appropriate guidance at the same time as nurses are exposed to deeper and more focused training and education around pain procedures.

The main limitation of this study was related to its monocentric design in a French culture. Thus, the generalizability of our results cannot be assumed. These findings should be confirmed by multicenter study on larger sample. We conclude that future research should focus on assessing the impact of such programs to manage incident and procedural pain more successfully and could consider both nurse and patient perspectives about the topic.

## Conclusions

This qualitative study regarding the barriers to venipuncture pain prevention underlines the gap between the fact that nurses are aware of the recommendations and its implementation in clinical practice. The finding suggests different points needing to be addressed. The improvement should be targeted to each respective unit considering the specificity of each setting, a training program focused on procedural pain, nursing involvement in organizational aspect of care, and engagement in writing protocols and leaflet for better patient information.

## Abbreviations

AC: Anne Chirac
APICIL: Association de Prévoyance Interprofessionnelle des Cadres et Ingénieurs de la région Lyonnaise
CC: Claire Chabloz
CyC: Cyrille Collin
ICVAP: implantable central venous access port
LM: Léa Monsarrat
MF: Marilène Filbet
MR: Murielle Ruer
PL: Philip Larkin
WR: Wadih Rhondali
